# Epidemiology of *Taenia saginata* taeniosis/cysticercosis: a systematic review of the distribution in East, Southeast and South Asia

**DOI:** 10.1186/s13071-020-04095-1

**Published:** 2020-05-07

**Authors:** Ramon M. Eichenberger, Lian F. Thomas, Sarah Gabriël, Branco Bobić, Brecht Devleesschauwer, Lucy J. Robertson, Anastasios Saratsis, Paul R. Torgerson, Uffe C. Braae, Veronique Dermauw, Pierre Dorny

**Affiliations:** 1 Institute of Parasitology, Vetsuisse Faculty, University of Zurich, Zurich, Switzerland; 2 Institute of Infection and Global Health, University of Liverpool, Liverpool, UK; 3 International Livestock Research Institute, Nairobi, Kenya; 4 Faculty of Veterinary Medicine, Ghent University, Merelbeke, Belgium; 5 Centre of Excellence for Food– and Vector–borne Zoonoses, Institute for Medical Research, University of Belgrade, Belgrade, Serbia; 6 Department of Epidemiology and Public Health, Sciensano, Brussels, Belgium; 7 Department of Paraclinical Sciences, Faculty of Veterinary Medicine, Norwegian University of Life Sciences, Oslo, Norway; 8 Veterinary Research Institute, Hellenic Agricultural Organization Demeter, Thermi, Greece; 9 Section of Veterinary Epidemiology, Vetsuisse Faculty, University of Zürich, Zürich, Switzerland; 10 One Health Center for Zoonoses and Tropical Veterinary Medicine, Ross University School of Veterinary Medicine, Basseterre, Saint Kitts and Nevis; 11 Department of Infectious Disease Epidemiology and Prevention, Statens Serum Institute, Copenhagen, Denmark; 12 Department of Biomedical Sciences, Institute of Tropical Medicine, Antwerp, Belgium

**Keywords:** *Taenia saginata*, Bovine cysticercosis, Beef tapeworm, Cestode, Foodborne pathogen, Taeniosis, East Asia, Southeast Asia, South Asia, Zoonosis

## Abstract

**Background:**

*Taenia saginata* is an important zoonotic parasite, causing taeniosis in humans and cysticercosis in bovines, the latter being a significant concern for the global beef industry. Many countries in East, Southeast and South Asia are experiencing rapid economic growth, and an increasing number of people in these countries are dependent on the livestock industry. Currently, however, an overview of the prevalence of *T. saginata* in this region is lacking. In this review, we analysed the available literature on *T. saginata* taeniosis and bovine cysticercosis for East, Southeast and South Asia.

**Methods:**

A systematic review was conducted, based on both published and grey literature. Articles published between 1990 and 2017 were mined for information on the occurrence, prevalence, and geographical distribution of *T. saginata* taeniosis and bovine cysticercosis in East, Southeast and South Asia.

**Results:**

The presence of *T. saginata* was described in 15 of 27 countries of the region, including Afghanistan, Cambodia, China, India, Indonesia, Japan, Lao PDR, Malaysia, Mongolia, Nepal, Pakistan, Philippines, South Korea, Thailand and Vietnam. The only country that reported an absence of *T. saginata* is Japan, although sporadic reports of imported cases and unconfirmed reports of autochthonous infections were identified. Nationwide surveys of taeniosis with systematic sample collection and high sample numbers were available for Cambodia, China, Lao PDR, and South Korea, although speciation of *Taenia* was not always performed. Regional prevalence of taeniosis and bovine cysticercosis in endemic regions ranged between 0.02–42.6%, and 0.76–46.7%, respectively. However, data for bovine cysticercosis were only available for five countries (Japan, Lao PDR, Mongolia, Pakistan and Vietnam).

**Conclusions:**

The data indicate a widespread occurrence of *T. saginata* throughout East, Southeast and South Asia. Identification of *Taenia* spp. in human infections was frequently not performed, leading to gaps in knowledge about the distribution of human tapeworm infections, mainly in regions where different human *Taenia* species co-occur. A high prevalence of *T. saginata* taeniosis and bovine cysticercosis may reflect insufficiencies in sanitation, limited health education standards, and insufficient food safety measures. Therefore, there is a need to improve local surveillance, notification, and overall control systems. 
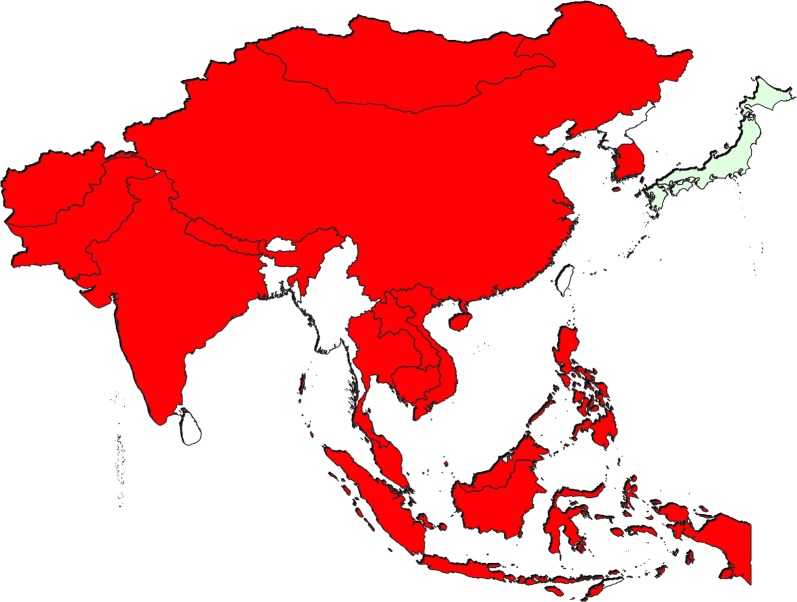

## Background

Large tapeworms parasitizing humans include the three *Taenia* species, *Taenia saginata* (beef tapeworm), *Taenia solium* (pork tapeworm), and *Taenia asiatica* (Asian tapeworm), all of which are prevalent in Asia and the Pacific [1]. *T. saginata* is considered the most common zoonotic tapeworm, with an estimated 60–70 million carriers globally [2]. Humans are the only definitive host, in which sexual reproduction of the adult tapeworm takes place in the intestine. The only intermediate host, bovines (such as cattle, buffaloes and yaks), harbour the larval parasitic stage in various muscle tissues (cysticercus, metacestode). Humans become infected by eating raw or undercooked meat containing viable cysticerci, which then develop to tapeworms in the intestinal tract.

Taeniosis is usually asymptomatic but mild symptoms, commonly associated with abdominal discomfort and independent migration of proglottids from the anus, have been commonly reported. Complications, such as appendicitis, have been reported to occur on rare occasions [3, 4]. Cysticercosis in bovines is usually not associated with clinical signs. Nevertheless, bovine cysticercosis may generate economic losses in regions where cattle are commercially farmed, due to the requirement for special handling (condemnation, freezing, and export restrictions) of infected carcasses, as legislated by meat hygiene regulations [5, 6].

Beef markets and trade have developed at a rapid pace in different Asian countries in recent years, with important implications for rural development, food security, human nutrition, and trade, and with a growing number of people involved in and dependent on the cattle industry [7, 8]. On the downside, the spread of taeniosis/cysticercosis is facilitated by poor hygiene, inadequate sanitation, the use of untreated or partially treated human waste in agriculture, lack of knowledge concerning the risks, and the consumption of raw or undercooked beef [9].

As a part of a EU-COST Action framework “CYSTINET” (www.cystinet.org) activity aimed at mapping global data on the distribution of *T. saginata* taeniosis/cysticercosis, this review focuses on the prevalence of the beef tapeworm in East, Southeast and South Asia, a fast-growing region with rich cultural, traditional and religious diversity.

## Methods

### Search strategy

We performed a systematic database review, complying with PRISMA guidelines [10], of published and publicly available literature for information on the occurrence, prevalence, and geographical distribution of *Taenia saginata* taeniosis and cysticercosis in East, Southeast and South Asia between 1990 and 2017 (Additional file 1). The following countries and territories were screened for available literature and data: Afghanistan, Bangladesh, Bhutan, Brunei, Cambodia, China (disambiguation of People’s Republic of China), the Democratic Peopleʼs Republic of Korea (North Korea), East Timor, Hong Kong, India, Indonesia, Japan, Lao People’s Democratic Republic (Lao PDR), Macau, Malaysia, Maldives, Mongolia, Myanmar (formerly Burma), Nepal, Pakistan, Philippines, the Republic of Korea (South Korea), Singapore, Sri Lanka, Taiwan (disambiguation of Republic of China), Thailand, and Vietnam.

### Database search and selection criteria

The following online databases were screened for publications: PubMed, ISI Web of Science, OpenGrey, OAIster, CABDirect, J-Stage, Asia journals online, WHO IRIS, Index Medicus for South-East Asian Region, China National Knowledge Infrastructure, and various regional databases and local thesis collections (webpages available in Additional file 2: Text S1).

The literature search was performed using the search terms (cysticerc* OR cisticerc* OR “C. bovis” OR taenia* OR tenia* OR saginata OR taeniosis OR teniosis OR taeniasis OR tenia OR taeniid OR cysticerque OR taeniarhynchus) AND (Afghanistan OR Bangladesh OR Bhutan OR Brunei OR Cambodia OR China OR East Timor OR Hong Kong OR India OR Indonesia OR Japan OR Lao OR Macau OR Malaysia OR Maldives OR Mongolia OR Myanmar OR Burma OR Nepal OR Korea OR Pakistan OR Philippines OR Singapore OR Sri Lanka OR Taiwan OR Thailand OR Vietnam).

Publications were included if published between January 1st, 1990 and December 31st, 2017, and contained information about *T. saginata* taeniosis and/or cysticercosis. Publications in all languages were included and external assistance in translation was required for one publication that was written in Japanese, but which was later excluded due to the absence of any prevalence data. Literature was excluded if: (i) only parasites other than *T. saginata* were reported; (ii) only countries and territories outside of the prescribed list were described; (iii) described data were not collected within the specified timeframe; (iv) only experimental data were reported; and (v) if data were duplicated from an earlier publication (e.g. same cases and numbers included in two different retrospective studies). Review articles were considered only if they contained unique data, that could not be retrieved otherwise. Full text articles were screened and data were extracted, including data period, province/district/locality (if possible with coordinates of the study site), number of total and positively tested cases, applied diagnostic methods used, and *Taenia* specification. Data without species identification were only included for regions where pig husbandry was presumed absent or banned due to cultural and religious traditions as clearly indicated by ethnicity of the local population. These results, however, are further discussed and should be interpreted with caution. For countries for which the previous approaches had not provided any record, the ‘Google Scholar’ search engine was consulted to identify documents containing *T. saginata* prevalence data using the search term “intestinal parasites” and the corresponding territory. These results are mentioned separately but included in the geographical mapping.

If available, prevalence data were recorded on a regional level. For prevalence at country/territory level, data from all the papers were combined, with prevalence and Wilson score confidence intervals calculated based on the reported numbers. Data analyses were performed in R version 3.5.2 [11]. Maps were generated using the open source software QGIS 3.0.1.-Girona (http://qgis.org) with 1:10m raster and vector maps retrieved from the *Natural Earth* public domain (http://www.naturalearthdata.com/downloads/).

## Results

**Fig. 1:**
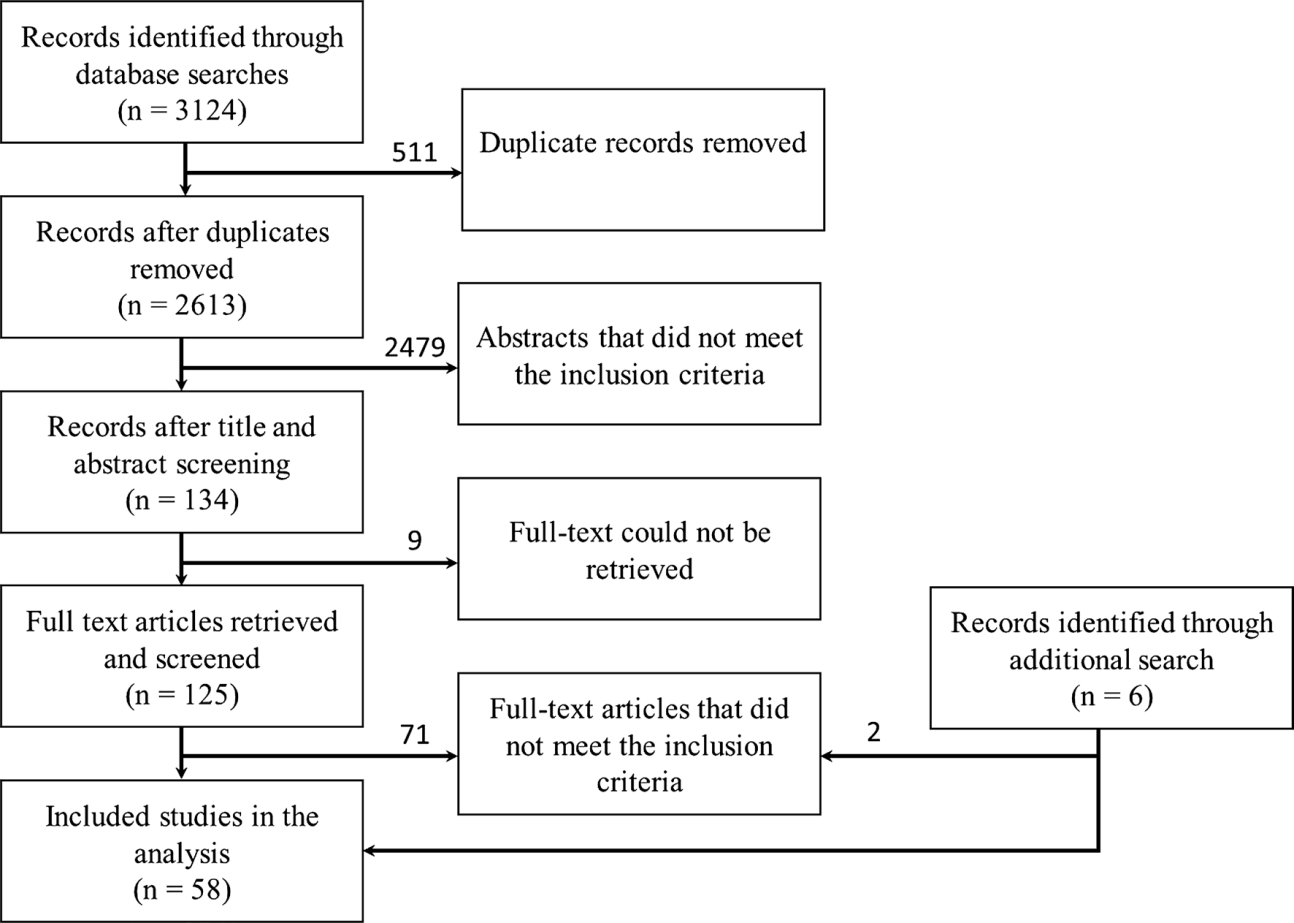
Flow diagram of the database searches according to PRISMA guidelines [10]

Out of 3124 initially screened hits, the final data search revealed 58 eligible publications containing data on *T. saginata* taeniosis or cysticercosis, of which 56 included information on the occurrence of human taeniosis, and six on bovine cysticercosis (Fig. 1). These documents included 47 original studies, 10 review articles, and 1 doctoral thesis, all of which included unique data. Data for 15 out of 27 countries and territories could be analysed. No data for the reported period could be retrieved for Afghanistan, Bangladesh, Bhutan, Brunei, East Timor, Hong Kong, Macau, Malaysia, Maldives, Myanmar, North Korea, Singapore, Sri Lanka and Taiwan. However, in the expanded literature search (by Google Scholar), data on the prevalence of *T. saginata* in Afghanistan (4 additional publications, of which 2 contained unique data) and Malaysia (2 additional publications) were identified.

### Taeniosis in humans

Data on the prevalence of taeniosis in East, Southeast and South Asia varied markedly across study sites, depending on dietary habits, husbandry practices, and the socioeconomic status (Table 1). All the studies included were based on initial identification of *Taenia* stages in faecal samples by microscopy. Tapeworm specification by copro-PCR was reported for Indonesia, Lao PDR, the Philippines, Thailand and Vietnam. In countries and territories where an absence of widespread pork eating habits or a legal ban of pork (mainly in Muslim communities) is present, such as Afghanistan and Pakistan, *T. saginata* was assumed to be the predominant *Taenia* species, if the data were collected from an indigenous population. Some authors discussed the species of *Taenia* based on the ethnicity, eating habits of the communities, and specific questionnaire data (relevant for India, Lao PDR, Mongolia, Nepal and Thailand).

**Table 1:** Reported prevalence of human *Taenia saginata* taeniosis in East, Southeast and South Asia

Country	Period	Group studied	Prevalence (%) (95% CI)	Range of regional prevalence (regional coverage)^a^	Reported species	References
Afghanistan	2011–2014	Soldiers (*n *= 110); hospitalized (*n *=1329); students (*n *=1869)	1.39 (1.03–1.87)	0.62–1.43 (+)	*Taenia* spp.	[52, 53]
Cambodia	2006–2011	Schoolchildren (*n* = 27716); adults (*n* = 7309)	0.45 (0.38–0.52)	0–2.34 (++)	*T. saginata* (90.5%); *T. solium* (9.5%)	[12, 13]
China	2001–2004	National survey on taeniosis and cysticercosis (*n* = 356629)	0.28 (0.26–0.30)	Nationwide survey (+)	*Taenia* spp.	[45, 54–57]
India	2004–2013	Children (*n* = 3992); adults (*n* = 362)	3.84 (3.29–4.46)	0.68–4.63 (−)	*T. saginata*	[29, 30, 58–60]
Indonesia	1996–2006	Residents (*n* = 2906)	4.68 (3.95–5.53)	0–22.50 (+)	*T. saginata*	[25, 61, 62]
Japan	1995–2010	na	0	na	*T. saginata*	[17, 18, 23]
Lao PDR	2000–2013	Residents (*n* = 58006)	1.56 (1.45–1.65)	1.56–11.50 (+)	*T. saginata*	[63–67]
Malaysia	2001; 2013	Schoolchildren (*n* = 111); residents (*n* = 110)	1.81 (0.58–4.88)	0.90–2.73 (−)	*Taenia* spp.	[68, 69]
Mongolia	1998	Adult farmers (*n* = 206)	0.49 (0.03–3.09)	(-)	*T. saginata*	[70]
Nepal	2007–2012;	Schoolchildren (*n* = 503);	4.37 (2.83–6.65)	1.75–4.71 (+)	*T. saginata*	[42, 71–73]
	2011–2012	Schoolchildren (*n* = 1704)	0.41 (0.18–0.88)	0.22–1.30 (−)	*Taenia* spp.	
Pakistan	2006–2014	Residents and schoolchildren (*n* = 5247)	7.01 (6.34–7.75)	0.21–12.35 (++)	*T. saginata*	[31–34, 74, 75]
Philippines	2005; 2011	Schoolchildren (*n* = 259), residents (*n* = 549)	33.71 (30.46–37.11)	15.10–42.57 (-)	*T. saginata*	[76, 77]
South Korea	1997; 2004 –2011	Residents (*n* = 782), national survey (*n* = 45832)	0.03 (0.02–0.05)	0.02–0.51 (−)	*T. saginata*; *Taenia* spp.	[78, 79]
Thailand	2004–2013	Residents (*n* = 1343), schoolchildren (*n* = 1920)	0.31 (0.16–0.58)	0.30–1.60 (+)	*T. saginata*	[41, 80–83]
Vietnam	2015	Residents (*n* = 342)	5.85 (3.70–9.03)	0.20–12.0 (++)	*T. saginata* (45.3%); *T. asiatica* (49.7%); *T. solium* (5.0%)	[14–16, 22]

^a^Regional coverage is indicated in parentheses:-, poor; +, < 50% of the national territory; ++, > 50% of the national territory); for details see Fig. 2 and extracted data in Additional file 2: Text S1

na, not available; CI, confidence interval

**Fig. 2:**
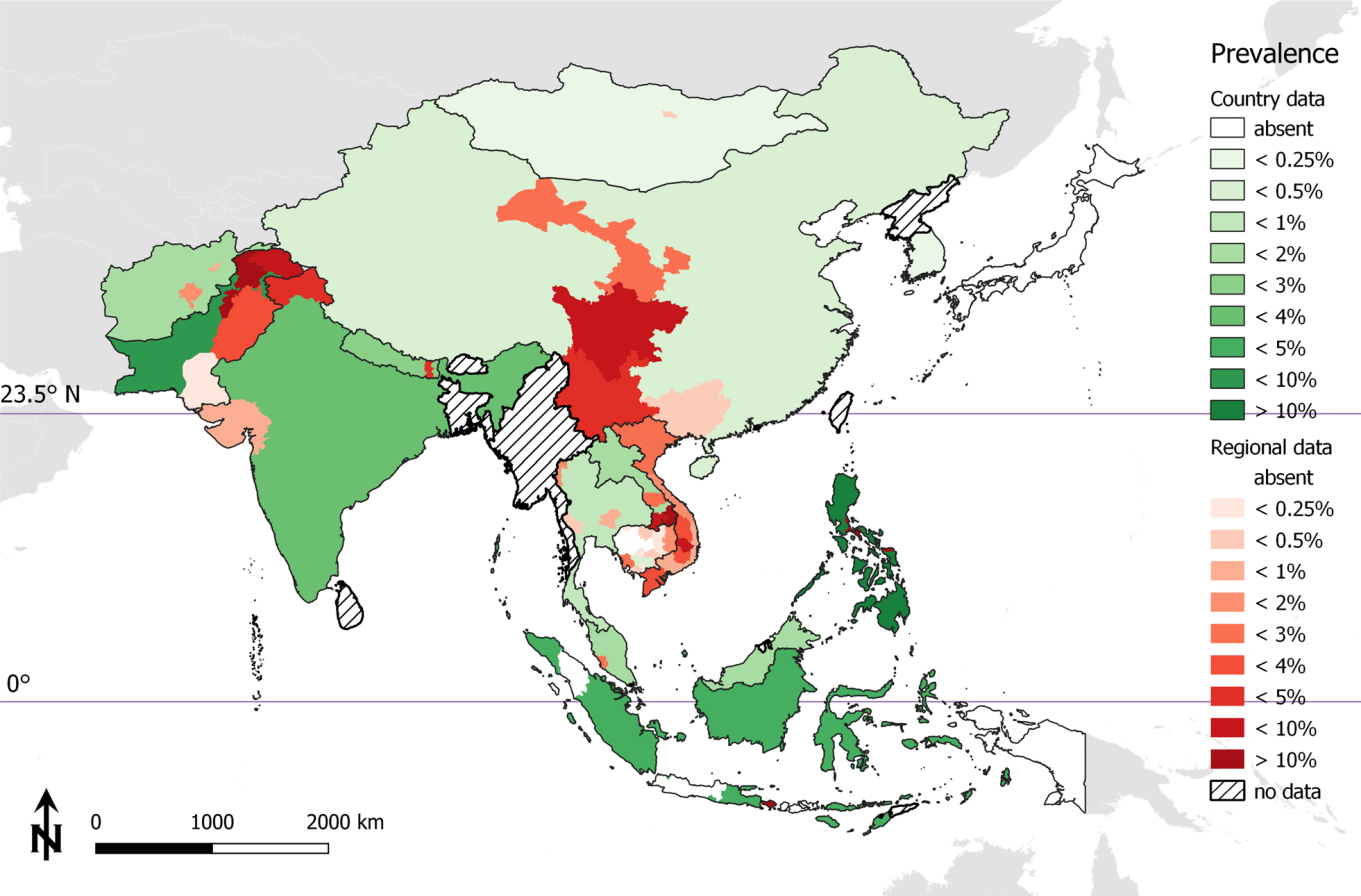
Assumed human *T. saginata* taeniosis in East, Southeast and South Asia

For Cambodia, where different zoonotic *Taenia* species co-occur, faecal examination on representative sample numbers (*n *=35,025) from schoolchildren and adults were performed [12, 13]. There, *Taenia* species specification by multiplex PCR (*cox*1 gene) and sequencing was performed on a subset of the positive samples (43 *Taenia* spp. positives in 2824 analysed samples) collected from different parts of the country, revealing that 90.5% of the taeniid eggs detected by coproscopy were *T. saginata* and 9.5% *T. solium*, whereas *T. asiatica* was absent [12]. Overall, this allows for an approximate prevalence estimation for *T. saginata* taeniosis on a countrywide scale. In Vietnam, the majority of the studies did not discriminate between the different *Taenia* species; however, molecular studies not designed to assess prevalence confirmed the co-occurrence of the different human *Taenia* species in the different provinces, with *T. asiatica* accounting for the most abundant species found in positive humans (49.7% of the positives; 95% confidence interval, CI: 41.7–57.7%), followed by *T. saginata* (45.3%; 95% CI: 37.5–53.4%), and *T. solium* (5.0%; 95% CI: 2.4–10.0) [14–16]. Unfortunately, only one prevalence study included molecular species identification, and this demonstrated a *T. saginata* prevalence of 5.85% (95% CI: 3.7–9.0) for the Central Highlands of Vietnam [16]. Data on the distribution of taeniosis indicate variable prevalence estimates of 0.2–12% for the different regions in Vietnam. Average prevalence of taeniosis per country and regional distribution (where available) is illustrated in Fig. 2 and collected reports are provided in Additional file 3: Data S1.

Surveys on a national level without determination of the *Taenia* species were reported for China and Nepal, whereas some indication about the distribution of *T. saginata* taeniosis can be retrieved from regional studies and population dietary habits. The studied population groups included schoolchildren, adults, the general population (all age groups) and from specific occupational groups (e.g. food handlers in Pakistan and farmers in Mongolia). Any hospital data based on patients suffering from gastrointestinal signs with stool examination and single case reports were excluded from the analysis because the prevalence could not be calculated and nor was any new evidence for the presence of the parasite provided. Nationwide surveys with systematic sample collections and high sample numbers were available for Cambodia, China, Lao PDR and South Korea (Table 1).

### Cysticercosis in cattle

**Fig. 3:**
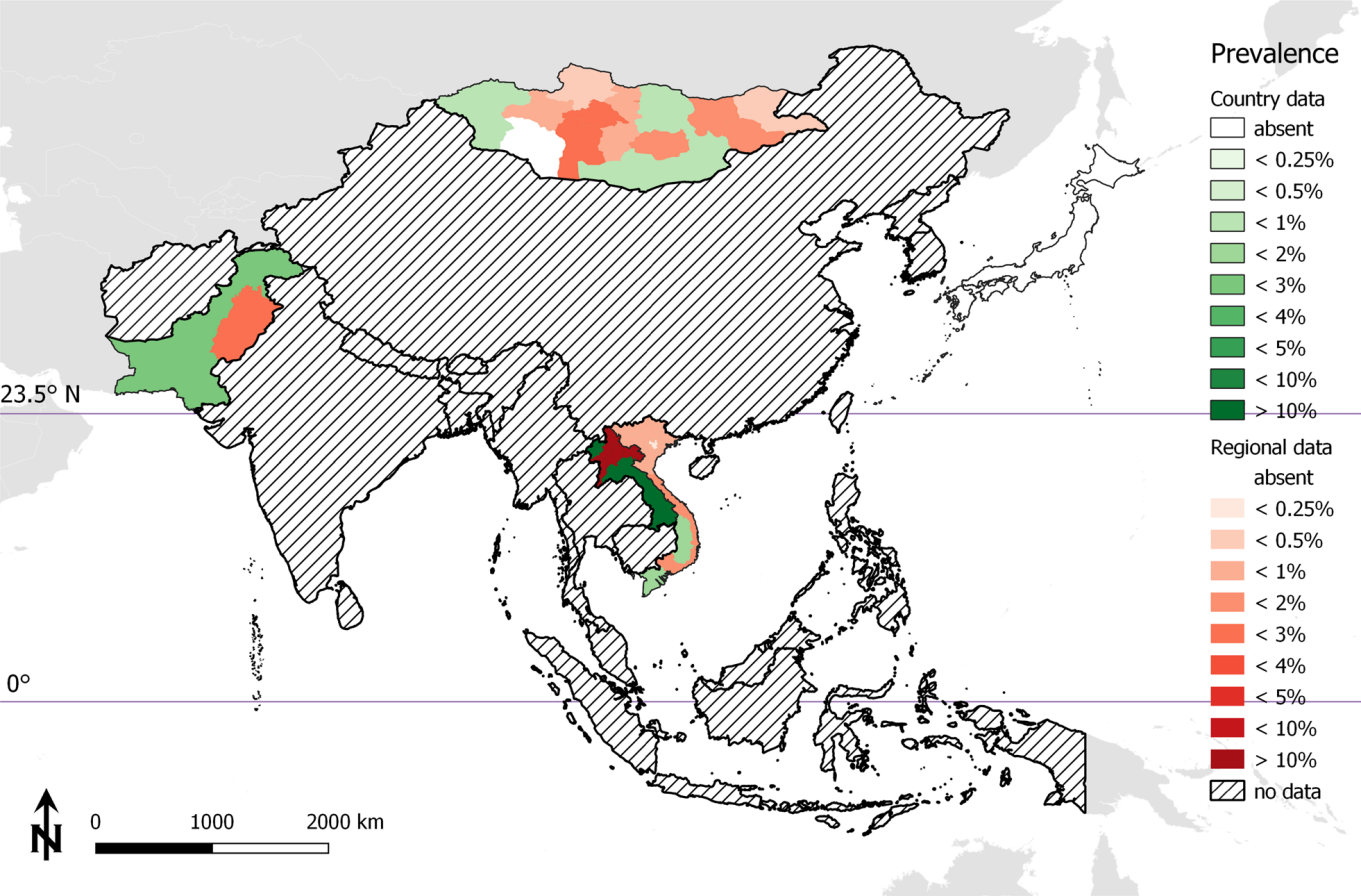
Bovine cysticercosis in East, Southeast and South Asia

Studies on the prevalence of bovine cysticercosis were available from Japan, Lao PDR, Mongolia, Pakistan and Vietnam (prevalence data are summarized in Table 2 and Fig. 3). Overall, Japan is considered to be free from autochthonous bovine cysticercosis based upon meat inspection results [17, 18]. A study in five provinces in northern Lao PDR conducted in 2006 describes the prevalence of *T. saginata* cysticercosis in cattle and buffaloes based on the detection of circulating antigens [19]. Overall, the prevalence of cysticercosis was 37.7% (95% CI: 33.9–41.8%) and 63.8% (95% CI: 58–69.2%) in buffaloes and cattle, respectively [19]. Cattle demonstrated a substantially higher prevalence than buffalo. The highest provincial prevalence was recorded in Xayabuly (69.4%; 95% CI: 61.5–76.4%), and the lowest was in Huapanh (36.8%; 95% CI: 31.5–42.4%).

**Table 2:** Reported occurrence of bovine cysticercosis in East, Southeast and South Asia

Country	Period	Animals tested	Animals positive	Prevalence (%) (95% CI)	Diagnostic technique	References
Japan	1999	na	na	Absent	MI	[17]
Japan	1995–2001	na	na	Absent	MI	[18]
Lao PDR	2006	904	422	46.7 (43.40–50.00)	Ag-ELISA	[19]
Mongolia	2002–2012	5760	44	0.76 (0.56–0.10)	MI	[20]
Pakistan	2012–2013	2400	73	3.04 (2.41–3.83)	MI	[21]
Vietnam	na	na	na	1.6	MI	[22]

Ag-ELISA, antigen enzyme-linked immunosorbent assay; CI, confidence interval; MI, meat inspection; na, not available

Davaasuren and colleagues reported bovine cysticercosis cases from 12 of 21 provinces in Mongolia based on discontinuous meat inspection reports for the years 2002–2012 [20]. A low prevalence of bovine cysticercosis was recorded, with 44 positives in 5760 carcasses (0.76%; 95% CI: 0.56–1.03%).

One study from Pakistan analysed the prevalence of cysticercosis in cattle and buffalo in Punjab based on routine meat inspection [21]. In 1200 cattle and 1200 buffaloes, 35 (2.92%; 95% CI: 2.07–4.08%) and 38 (3.17%; 95% CI: 2.28–4.36%) positive carcasses were detected, respectively. There were no significant differences between the regions and between the two bovine species.

Based on meat inspection reports, a current review describing the status of cysticercosis in cattle in Vietnam demonstrates a general infection rate of 1.6%, with 0.03–2.17% at Hanoi abattoirs, 0.5–1.4% in the north, 1.9–2.2% in the centre, and 1.6–1.8% in the south of the country [22]. Unfortunately, exact animal numbers could not be extracted from publications.

## Discussion

This review summarizes the occurrence of human *T. saginata* taeniosis and bovine cysticercosis in East, Southeast and South Asia from 1990 to 2017. Although publications from almost 30 years were screened, information retrieved does not allow for a temporal analysis of changing infection dynamics (see Additional file 3: Data S1). However, the data demonstrate a widespread distribution of tapeworm infections throughout the region, with the only country reporting absence of autochthonous *T. saginata* being Japan, based upon the absence of bovine cysticercosis cases detected during meat inspection [17, 18]. Despite this assumption, cases of taeniosis are sporadically reported as being imported in travellers and there are some unconfirmed reports of patients that appear to have been infected in Japan [23]. Given the data described in this review indicating the presence of *T. saginata*, it is likely that this parasite is endemic throughout Asia.

### Co-distribution of *Taenia* spp.

Many countries in East, Southeast and South Asia are characterised by a plurality of distinct religious, native ethnic and linguistic groups. Within the context of different cultural and religious practises, the three different human *Taenia* species, i.e. *T. saginata*, *T. asiatica* and *T. solium*, have all been demonstrated to be circulating in the region [24]. For example, on the Indonesian Archipelago, which is highly influenced by an ethnic diversity and religious pluralism within a Muslim-majority population, all three of these human *Taenia* species are prevalent [25, 26]. Studies from Cambodia and Vietnam with considerable sample size numbers and high regional coverage, also reported the co-existence of the three different *Taenia* spp. parasitizing humans [12–14, 22, 27].

Not all eligible studies and reports, however, provide data by species. Although, it is possible to make some assumptions of species distribution based upon religious prohibitions on pork or beef consumption, this is of course accompanied by the caveat that diversity both in religion and adherence to religious doctrine, does not rule out the presence of other *Taenia* spp. In predominantly Muslim countries, such as in Pakistan and Afghanistan, where slaughtering and consumption of pork is not generally practised, we assume that the vast majority of taeniosis infections are *T. saginata*. Nevertheless, in some countries and territories, it is likely that pig production occurs in low numbers, with a consequent minor number of recorded *T. solium* infections. In contrast, in India, little information on *T. saginata* is available due to a ban on the slaughter of cows for religious reasons [28], although beef and carabeef consumption may be more widespread in the predominantly Muslim states in the north of India. Indeed, *T. saginata* has been detected at border regions to Pakistan, like Kashmir State [29] and Gujarat [30]. Likewise, studies from the border areas in Pakistan have demonstrated the presence of *T. saginata* taeniosis and cysticercosis [31–38]. Studies in schoolchildren from southern parts of India also indicate a high prevalence of *Taenia* spp. (5% in Tamil Nadu and 3.58% in Puducherry, respectively) [39, 40]. However, these records were excluded from the list as they did not provide or discuss species identity.

### Culinary risk factors

In general, cultural dietary habits of raw or undercooked beef in traditional dishes are common risk factors of infections in the study region, such as ‘*bo tai chanh*’ (raw or rare beef in lime juice salad) in Vietnam, ‘*lawar*’ with ‘*tuak*’ (raw beef with palm wine) in the traditional village communities in Bali/Indonesia, ‘*buuz*’, ‘*huushuur*’ and ‘*bansh*’ (dumplings prepared with minced and seasoned raw beef) in Mongolia, ‘*yukhoe’* (a raw meat dish that resembles a steak tartare) in Korean cuisine, and ‘*phla nuea*’ and ‘*yam neua*’ (variations of Thai beef salads) in Thailand [20, 25, 27, 41].

In many countries within the region of interest, consumption of raw pork is also popular, increasing the risk for *T. solium* and *T. asiatica*. For example, in Nepal, domestic pork is traditionally eaten by the ethnic group of ‘Aadi basi’ (corresponds to the biggest studied population in [42]). Raw pork is commonly consumed as part of sacrificial ceremonies amongst Tai Dam communities in northern Lao PDR [43]. Wild boar is traditionally hunted and eaten by ‘Magars’, but a strain bred from wild boar is now raised in captivity and used for meat that is increasingly popular among ‘Prahari’ ethnicities (largest ethnic group in Nepal) and other groups that do not traditionally eat pork. Nepalese Hindus (similar to Indian Hindus) do not slaughter cattle; this restriction does not, however, extend to buffaloes, which are consumed in some Hindu communities. Hence, the prevalence data for Nepal (0.22% in school children and 1.3% in a hospital population) must be interpreted with care in terms of likely speciation. Likewise, in Bhutan, which can be compared to Nepal, the only available data on *Taenia* spp. indicate an overall low prevalence of 0.17% [44]. There is an absence of studies with species identification for southwest China, and the prevalence and incidence of human taeniosis remains unknown despite local people choosing to eat undercooked beef, raw pork, and raw pig liver mixed with sour sauce and salted garlic [45].

### Distribution of *T. saginata* taeniosis and cysticercosis

Despite the limitations of the data in terms of speciation, this review identified widespread occurrence of taeniosis in humans, with a marked variation in prevalence across the regions as would be expected due to the variations in socioeconomic status, cultural and animal husbandry practises across the region.

Data on bovine cysticercosis are, in general, rare. Although excluded from our analysis because of outdated meat inspection reports, data on cysticercosis from cattle in Bali (Indonesia) were reported in 1989 from the Stockbreeding Service Unit Denpasar [46]. There, routine meat inspection in 1988 reported that 674 of 34,887 cattle tested positive for bovine cysticercosis (1.93%; 95% CI: 1.79–2.08%). However, the current high detection rate of taeniosis in humans suggests that the actual prevalence in cattle is probably higher. Indeed, the sensitivity of general meat inspection protocols is considered below 30% [47]. Based on slaughterhouse reports, prevalence estimates are available for Mongolia (0.76%) and Vietnam (1.6%) [20, 22]. However, serological testing based on the detection of circulating antigens [48] in Lao PDR showed that 46.4% of cattle were infected, indicating an underestimate of bovine cysticercosis based on meat inspection reports [19]. Lao PDR is one of the lowest-income countries in the Southeast Asian region, and has a predominantly rural-based agricultural economy. Cattle and buffalo production contributes significantly to Lao PDR’s rural economy, with approximately 31% and 48% of households raising cattle and buffalo, respectively [49]. This represents a risk for parasite transmission due to home-slaughtering and lack of meat inspection and control [49].

This article complements recent reviews on the distribution of *T. saginata* taeniosis/cysticercosis in central and western Asia and the Caucasus [50], and on the epidemiology of porcine cysticercosis in East and Southeast Asia [51]. Together with these companion articles, it demonstrates the widespread distribution of taeniosis and cysticercosis throughout the continent, with regional variations in the prevalence. The regional characteristics of different local lifestyles and the co-occurrence of different soil-transmitted parasites and neglected tropical diseases, among the different human *Taenia* species, reflect the particular importance for sustainable monitoring and implementation of control programmes (discussed in [24]). However, the collected data illustrate that detailed national prevalence data are missing.

## Conclusions

Despite the large diversity of cultural, traditional, and behavioural practices, *T. saginata* remains widespread throughout East, Southeast and South Asia. Many publications focus on the prevalence of soil-transmitted helminths and other neglected tropical diseases, but specification of the three co-occurring human *Taenia* species is rarely conducted. Regional prevalence data are lacking for many parts of East, Southeast and South Asia. Nevertheless, the presented data reveal a large range of prevalence rates for the different countries and territories, allowing for an overview of the ongoing situation in the region. Thus, the abundant prevalence of *T. saginata* in these low-income countries indicates gaps in environmental, food, and personal hygiene. Actions to control and prevent *T. saginata* infections should include improved sanitation, health education, food safety measures with improved and standardized diagnostic tests, and reporting of infections at the species level. These interventions can be implemented in a One Health approach accounting for human, animal, and environmental health.

## Supplementary information

**Additional file 1:** PRISMA 2009 checklist.

**Additional file 2: Text S1.** Databases for the literature research.

**Additional file 3: Data S1.** Data analysis of collected reports.

## Data Availability

All references found eligible in our literature review are included in the article.
